# Harnessing Flowering Bund Plants Through Ecological Engineering to Improve Biological Control of Tungro Virus Vectors in Indonesian Rice Fields Agroecosystem

**DOI:** 10.1155/ijfo/2476370

**Published:** 2025-08-05

**Authors:** Nur Rosida, Elisurya Ibrahim, Wasis Senoaji, Effi Alfiani Sidik, Ani Mugiasih, Khaerana, Rudi Tomson Hutasoit, Firmansyah, Raden Heru Praptana, Sri Sudewi, Andi Nasruddin

**Affiliations:** ^1^Research Organization for Agriculture and Food, National Research and Innovation Agency (BRIN), Cibinong, West Java, Indonesia; ^2^Faculty of Agriculture, Universitas Hasanuddin, Makassar, South Sulawesi, Indonesia

**Keywords:** ecological engineering, flowering bund plants, green leafhoppers, natural enemies, tungro disease

## Abstract

Ecological engineering (EE) has emerged as a promising strategy for managing insect pests in rice ecosystems by reducing dependency on chemical pesticides. However, the survival of parasitoids and predators in rice habitats is often limited due to a lack of refuge and food sources. While pesticides remain the primary method used by farmers to control green leafhoppers (GLHs), the main vectors of the tungro virus, their overuse poses serious risks to both environmental and human health. This study was aimed at evaluating the impact of EE on the population dynamics of GLHs and their natural enemies, the suppression of tungro virus infection, and the maintenance of rice yield. Field experiments were conducted in Sidrap, Indonesia, across three dry seasons (2016, 2017, and 2021) using three treatments: EE with biopesticide (T1), prophylactic insecticide use (T2), and untreated control (T3). The GLH population was significantly lower in T1 compared to T2 and T3, while natural enemy abundance (e.g., spiders, parasitoids, and predatory beetles) was highest in T1, particularly during later growth stages. Tungro incidence was lowest in T1, moderate in T2, and highest in T3, exceeding 13% in control plots. Despite differences in pest pressure and virus infection, rice yield did not differ significantly among treatments, with the highest yield (≈10–11 t/ha) recorded in 2021. These findings suggest that habitat manipulation through EE can suppress tungro vectors, enhance natural enemy populations, and sustain rice productivity without relying on chemical inputs.

## 1. Introduction

Rice (*Oryza sativa* L.) is the staple food for many people, particularly in Southeast Asia, the West Indies, the Middle East, and Latin America [1–3]. A significant challenge to global rice production is the persistent loss of crops due to plant diseases, including rice blast, sheath blight, bacterial blight, and notably, vector-borne rice virus diseases such as tungro [3, 4]. Tungro disease, caused by the rice tungro bacilliform virus (RTBV) and the rice tungro spherical virus (RTSV), is one of the most destructive rice diseases in Indonesia [5–9]. These viruses are transmitted in a semipersistent manner by the green leafhopper (GLH) (*Nephotettix virescens*) [10–12], emphasizing the importance of vector control in tungro management.

Insecticides have long been used to manage GLHs, but their effectiveness is limited due to asynchronous cropping patterns and the high mobility of the vector [9, 13–15]. Moreover, excessive insecticide use has triggered secondary pest outbreaks, pest resistance, and the collapse of natural enemy populations, reducing ecological resilience in rice fields [16–20].

An alternative and sustainable strategy is ecological engineering (EE), which enhances the abundance and efficacy of natural enemies by diversifying rice field habitats [21–24]. EE includes habitat manipulation such as planting nectar-rich flowering plants on rice bunds to supply natural enemies with essential resources like nectar [25, 26], pollen [27, 28], shelter [29, 30], and alternative prey or hosts [31, 32]. Bunds, typically unutilized in Indonesian paddy fields, offer spatial opportunities for implementing EE practices [1, 33]. Prior studies such as Ali et al. [1], Alves et al. [34], Hunt et al. [35], and Snyder [36] have shown that such interventions can improve natural enemy longevity, fecundity, and pest suppression.

In addition to habitat manipulation, biopesticides based on entomopathogens and botanical extracts represent a promising alternative to synthetic chemicals, especially under evolving food safety regulations [37]. Among the most effective microbial control agents are entomopathogenic fungi (EPFs), particularly *Beauveria bassiana* and *Metarhizium anisopliae*, which are widely used due to their pathogenicity against a broad range of insect pests. These fungi naturally occur in soil and are known to cause muscardine diseases in arthropods [38, 39]. Furthermore, botanical extracts such as those derived from *Andrographis paniculata* (commonly known as sambiloto) have shown insecticidal, antifeedant, and repellent properties against various rice pests, including the GLH (*N. virescens*) [40, 41].

Despite previous efforts in EE, several knowledge gaps remain. For example, the study by Ibrahim and Mugiasih [42] assessed the diversity of pests and natural enemies in rice ecosystems with and without EE. However, it lacked a true control treatment—plots that received neither insecticides nor flowering plants. In addition, their study was conducted at different geographic coordinates and did not evaluate the interactions between pest populations, virus incidence, and rice yield across multiple growing seasons.

This study offers a novel contribution by evaluating the combined use of flowering bund plants and biopesticide application as an integrated EE treatment, in comparison with a prophylactic insecticide use and an untreated control. These treatments were assessed for their effects on GLH populations, natural enemy dynamics, tungro symptom expression, and rice yield across three dry seasons (2016, 2017, and 2021). Considering the above, the study aimed to evaluate the effectiveness of EE, combining flowering bund plants and biopesticide application, in managing GLH populations, increasing the abundance of natural enemies, suppressing tungro virus incidence, and maintaining rice yields.

## 2. Materials and Methods

### 2.1. Research Sites

The research was conducted in the rice field experiment of Tungro Disease Research Station, Agricultural Research and Development in Sidrap (3°51⁣′00⁣^″^ S, 119°50⁣′00⁣^″^ E), Indonesia. Approximately 90% of the land in this area is cultivated with rice, and it is considered a tungro-endemic region.

### 2.2. Preparation of Biopesticide

The dry formulation of the EPF *M. anisopliae* followed the method of Reddy et al. and Pertiwi et al. [39, 43] with slight modifications, comprising 40 g spore biomass flour, 20 g kaolin, 20 g zeolite, and 20 g corn flour. *M. anisopliae* cultured on corn media was cold-dried and stored at 5°C for 12 days. The dried culture was ground and sieved to obtain spore biomass powder, which was then mixed with carrier materials (kaolin, zeolite, and corn flour) and sambiloto (*A. paniculata* Nees) leaf extract [37, 41].

### 2.3. Experimental Design

**Figure 1:**
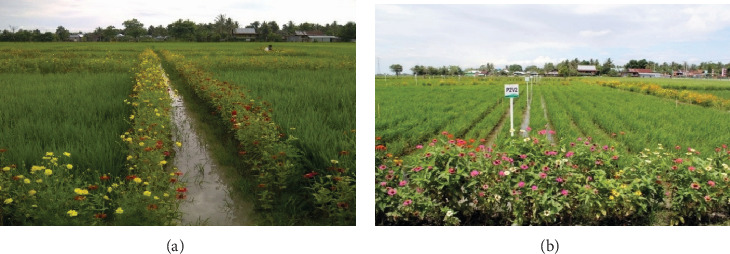
Flowering bund plants (zinnia, marigold, and cosmos) cultivated on the bunds of rice fields to offer resources for biocontrol agents during Dry Season (a) 2016 and (b) 2021, Lanrang (Sidrap).

The experiment was conducted during the dry seasons of 2016, 2017, and 2021. It consisted of nine individual plots, each bordered by bunds and arranged into three blocks. Treatments were assigned using a randomized complete block design (RCBD) with three replications (*n* = 3). Each plot measured 10 × 10 m and was separated by bunds to minimize interference from treatments. Three treatments were applied: (T1) EE using flowering bund plants combined with biopesticide applications, (T2) prophylactic insecticide applications, and (T3) untreated control. In T1, the bunds surrounding each plot were planted with flowering plants (*Cosmos* spp., *Zinnia* spp., and *Tagetes* spp.), and the rice crop was sprayed with a biopesticide composed of the EPF *M. anisopliae* and *A. paniculata* leaf extract (Figure 1). These agents were selected based on previous studies demonstrating their efficacy against rice pests, including planthoppers and leafhoppers, and their compatibility with ecological approaches [37, 39, 41, 44, 45]. T2 reflected local farmer practices, with fallow bunds and prophylactic application of a synthetic insecticide containing thiamethoxam (20%) and chlorantraniliprole (20%). T3 served as a control with fallow bunds and no pesticide application.

The flowering plants were sown on seedbeds 20–30 days prior to rice transplanting and transplanted to the bunds 10 days after sowing at a spacing of 20 × 20 cm. Bunds were prepared with manure 1 week before planting. Rice cultivars Inpari 9 Elo/Inpari 36 Lanrang were transplanted 19–21 days after sowing at 25 × 25 cm spacing. Crop management followed conventional local practices, including fertilization with 100 kg/ha urea and 300 kg/ha Ponska applied in three stages.

The biopesticide was prepared with a conidial concentration of 2 × 10^6^ spores/g (10 g) of *M. anisopliae* dry powder and 4 g/L of *A. paniculata* leaf extract, mixed with a surfactant (Tween 1 mL/L). Biopesticide applications were conducted weekly from 1 to 8 weeks after transplanting (WAT), typically in the late afternoon.

Insecticide applications in T2 were conducted in the seedbed and applied three times after transplanting during each season. This reflects the prophylactic spraying practices commonly adopted by local farmers. Insecticides containing neonicotinoid active ingredients are commonly used by farmers, who often apply them at regular intervals without considering pest threshold levels [46, 47]. The frequency of application tends to increase when visible crop damage is perceived as severe, largely driven by the fear of yield loss. As a result, pest population monitoring is rarely prioritized in pest management decisions. Similar patterns of prophylactic insecticide use—driven more by perceived threat than by evidence-based thresholds—have also been reported in Southeast Asian rice farming systems [1, 47–51].

### 2.4. Sampling of Insect Pests and Damage

#### 2.4.1. Sweeping Net Sampling

Insect samples were collected for each treatment during the maximum tillering stage of rice. Natural enemies and tungro vectors (GLHs) were captured using a sweeping net, making 10 sweeps with double swings in two diagonal directions within each observation plot [52]. The insects were placed in clear plastic bags (1 kg volume) containing cotton balls treated with 70% alcohol. Each bag was labeled with information about the location (village, subdistrict, and district), rice variety, plant age, and date of observation. The samples were then transported to the laboratory for sorting and counting the number and types of predators and GLHs (nymphs and adults) collected per plot.

#### 2.4.2. Tungro Incidence Percentage

The incidence of tungro was assessed by counting the number of plants showing symptoms of tungro virus infection (on a scale of 9) and dividing this number by the total number of plants in each observation plot. This result was then multiplied by 100% to obtain the percentage of plants infected with the rice tungro virus (RTV). The evaluation was based on the standard evaluation system [53]:

 $$\begin{array}{c} \%\inf.=\frac{\text{Number of plant infected}}{\text{Number of plant evaluated}}\times 100\%. \end{array}$$

#### 2.4.3. Rice Yield

The rice yields from each test plot were weighed and recorded in kilograms per hectare.

#### 2.4.4. Statistical Analysis

The effects of treatments on predator abundance, parasitoids, tungro incidence, and rice yield were analyzed using one-way ANOVA and Tukey's Honest Significant Difference (HSD) test. Data were transformed, if necessary, to achieve homogeneity using logarithmic or arc sine transformations before analysis. In addition, when the data value is 0, the transformation is done with ln (*n* + 1). All data were analyzed using SPSS software Version 21.0.

## 3. Results

**Figure 2:**
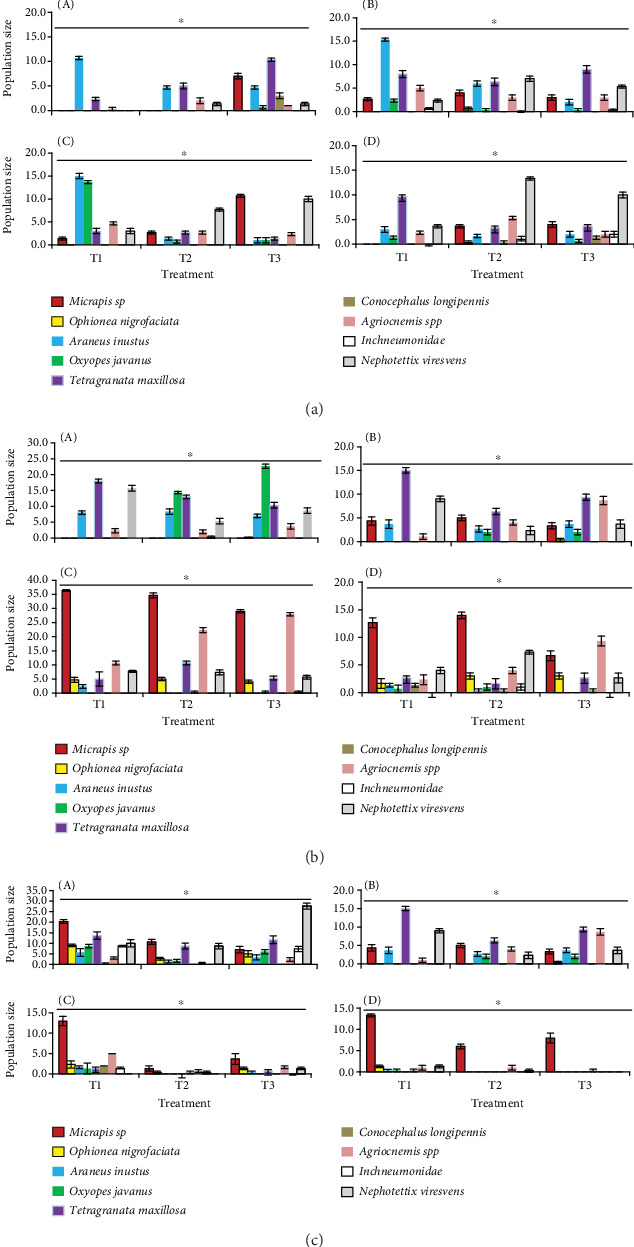
Impacts of treatments on the abundance of GLH (*Nephotettix virescens*) and natural enemies in a rice field, Sidrap, Indonesia. (a) Dry Season 2016; (b) Dry Season 2017; (c) Dry Season 2021. (A) 2 WAT; (B) 4 WAT; (C) 6 WAT; (D) 8 WAT. T1 = flowering plants grown on rice bunds, T2 = prophylactic insecticide use, and T3 = control. ∗ indicates a significant difference among treatments at the 5% significance level. The error bar indicates standard error. GLH = green leafhopper, WAT = week after transplanting.

The results showed that across all seasons and observation weeks (2, 4, 6, and 8 WAT), the population of GLHs was significantly lower in T1 (ecoengineering plots) than in T2 and T3 (Figure 2). Consistently, T3 exhibited higher GLH populations throughout the observation periods, highlighting the vulnerability of untreated crops to pest infestations. Across the seasons, T1 consistently demonstrated lower GLH populations compared to the control (T3), particularly in the later stages (6 and 8 WAT). This finding suggests that flowering plants enhance the ecosystem's ability to control pest populations naturally. For instance, in the 2021 season (Figure 2c), GLH populations under T1 were significantly reduced at 6 WAT compared to T3, indicating the cumulative positive effect of natural enemies over time (Figure 2c, A). The data also revealed that T2 initially reduced GLH populations effectively, as observed at 2 and 4 WAT (Figure 2c, A,B). However, a significant increase in GLH numbers was noted at 6 and 8 WAT, often nearing or surpassing those in T3. This rebounding effect could be due to several factors, including developing insecticide resistance and disrupting natural enemy populations. For example, in the dry season of 2017 (Figure 2b, A–D), GLH populations in T2 surged significantly at 8 WAT, nearly matching the levels observed in the untreated control (T3) (Figure 2b, D).

In contrast, natural enemy abundance (e.g., spiders, parasitoids, and predatory beetles) was consistently higher in T1, especially during the later stages of crop growth (Figure 2). This increased presence of natural enemies likely contributed to the lower GLH populations observed in T1. In the 2021 season (Figure 2c), the abundance of natural enemies in T1 was significantly higher at 6 and 8 WAT, demonstrating the positive impact of habitat enhancement on biological control agents (Figure 2c, C,D). In contrast, there was a decrease in natural enemies in the T2 treatment, especially in the early weeks (Figure 2c, A,B). This reduction can compromise the long-term stability of pest control as the season progresses. For example, in the 2016 season (Figure 2a), natural enemy populations in T2 were significantly lower throughout the observation periods compared to T1, indicating the detrimental effects of insecticides on nontarget organisms. T3 supported fewer natural enemies than T1 but more than T2, reflecting the absence of supportive and disruptive factors (Figure 2a, A–D).

Spiders, consisting of four species, were the rice fields' most common predators of GLHs. The predator population was most prominent in the T1 treatment, dominated by the lady beetle, *Micraspis* sp. (Figure 2c, A,B). However, the number of *Micraspis* sp. was not significantly different from that of spiders at 6 and 8 WAT. In addition to predators, parasitoids belonging to the family Ichneumonidae were also found in all treatments, with the highest numbers observed in T1 and T3 at 2 and 4 WAT. In T2, however, parasitoids were deficient at 6 and 8 WAT. Overall, the number of natural enemies in T2 involved insecticide application gradually decreased as the season progressed, except for *Micraspis* sp., which remained stable in all three treatments.

At 6 and 8 WAT, GLHs were no longer found in the T1 and T2 treatments, which differed from the control plot (T3). Similarly, the population of each natural enemy gradually decreased at 6 and 8 WAT, with natural enemies being almost nonexistent by 8 WAT, except for *Micraspis* sp. The highest number of *Micraspis* sp. was observed in the T1 treatment compared to the other treatments (Figure 2c, C,D).

**Figure 3:**
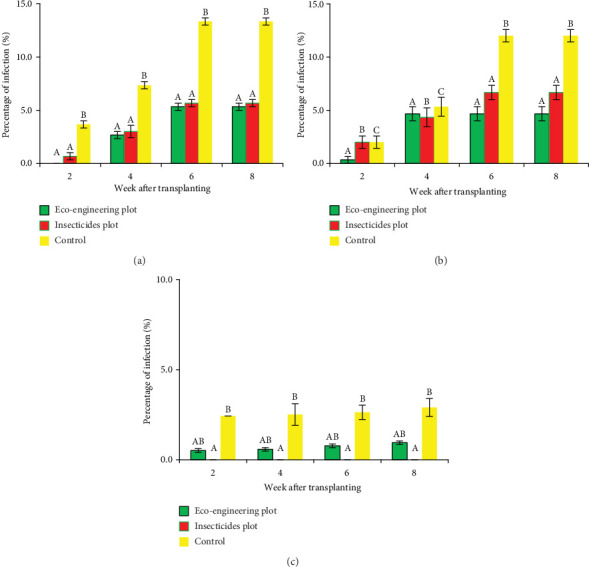
Effect of treatment on damage symptoms due to tungro virus attack in Lanrang rice fields, Sidrap, Indonesia. (a) Dry Season 2016; (b) Dry Season 2017; (c) Dry Season 2021. The mean values are shown here. Data were taken from 100 randomly chosen hills from each experimental plot—bars with letters that differ significantly at the 5% level. The error bar displays the default error.

The presence of flowering plants on paddy field bunds impacted the incidence of tungro disease. Across all years, T1 consistently had the lowest percentage of infected hills, followed by T2, while T3 (control) had the highest infection rates—reaching over 13% at 6–8 WAT in some years (Figure 3). The statistical analysis confirmed these differences (*p* < 0.05).

**Figure 4:**
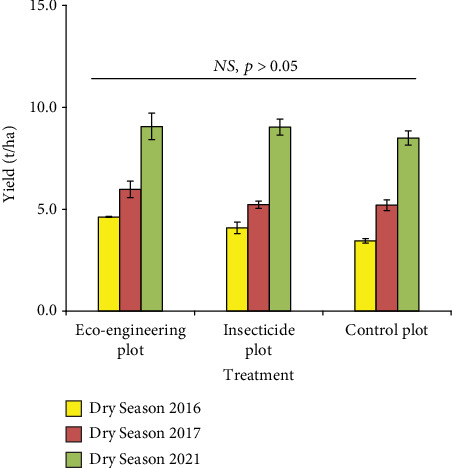
The effect of treatments on rice yields over several years. The Inpari 36 cultivar was used during the dry seasons of 2016, 2017, and 2021. *NS* indicates nonsignificant at the 5% level of significance. Error bars indicate standard errors.

Despite the differences in pest pressure and tungro incidence, there were no significant differences in rice yields (*p* > 0.05) among treatments. All treatments yielded similarly during each of the three seasons, with the highest yield (≈10–11 t/ha) observed in 2021 (Figure 4).

## 4. Discussion

*N. virescens*, commonly known as the GLH, is capable of feeding on various rice cultivars with differing resistance levels [54]. The density of *N. virescens* populations plays a crucial role in the development and spread of tungro disease. The severity of tungro outbreaks can vary significantly depending on environmental conditions. Besides vector abundance, other factors such as the widespread cultivation of susceptible rice cultivars and asynchronous planting schedules are believed to contribute to the occurrence of tungro epidemics [55].

Natural enemies are organisms that suppress the development of plant pests and diseases. Their use as biological control agents represents a promising strategy to reduce crop losses while meeting the increasing demand for high-quality, healthy, and environmentally friendly agricultural products. These ecofriendly products offer greater market value and competitiveness, particularly in international trade, due to their safety and sustainability attributes. Rice field ecosystems are often considered ecologically unstable and susceptible to pest outbreaks, especially under intensive monoculture practices. However, studies have reported that rice fields can harbor more than 700 insect species, including a wide array of predators and parasitoids that contribute to natural pest regulation [45, 56]. In systems with high insect biodiversity, the low incidence of planthopper pests has been attributed to strong ecological interactions such as predation and parasitism, which enhance the biological control potential of rice agroecosystems. Ecosystem stability in rice cultivation can be enhanced through proper management of species interactions, particularly through the adoption of integrated pest management (IPM), which promotes the reduction of synthetic pesticide use and emphasizes biological control. When natural enemies function effectively, they contribute to a more balanced and resilient agroecosystem [57, 58].

Natural enemies such as predators, parasitoids, and entomopathogens can effectively control GLH populations and support sustainable rice production. It has been reported that planthoppers are attacked by at least 79 natural enemies, including 34 parasitoid species, 37 predators, and eight EPFs [59]. Additionally, 27 predator species belonging to 16 insect families have been documented in rice agroecosystems [60]. However, the widespread adoption of high-yielding cultivars to meet food demands in countries like Indonesia has often been accompanied by the overuse of insecticides, which disrupt natural enemy populations and lead to pest resurgence [61, 62].

In the present study, we demonstrated that altering rice landscapes through EE—specifically by planting nectar-rich flowering plants on bunds and applying biopesticides (a combination of *M. anisopliae* and *A. paniculata* extract)—significantly enhanced the abundance of natural enemies, suppressed GLH populations, and maintained rice yield.

In Indonesia, rice production areas are typically fragmented into small plots, with each plot commonly managed by individual farmers. These plots are separated by bunds—raised ridges that function as irrigation boundaries and are usually left fallow. This landscape configuration offers a practical and underutilized opportunity for EE interventions. By converting these fallow bunds into habitats through the planting of nectar-rich flowering species, such as *Tagetes*, *Cosmos*, and *Zinnia*, our study enhanced the abundance of natural enemies while integrating seamlessly with existing farming layouts. This approach demonstrates that EE can be implemented without requiring major changes in land ownership or field infrastructure, which is particularly important for farmer adoption in smallholder rice systems.

In our study site, bunds typically left fallow were planted with marigold (*Tagetes* spp.), cosmos (*Cosmos* spp.), and zinnia (*Zinnia* spp.) (Figure 1). These plants provided critical resources such as nectar, pollen, and shelter to support the development and reproduction of beneficial arthropods. Nectar, in particular, supports parasitoid longevity, fecundity, and population growth [1, 63, 64]. Our findings showed that the presence of flowering bund plants increased the populations of predators and parasitoids in treatment T1, with the most substantial effects observed at 6 and 8 WAT (Figure 2). This result supports previous research by Zhang et al. [65], who found that habitat diversification improves biological control services in rice fields. GLH populations were consistently lower in T1 than in T2 and T3, with T2 initially reducing GLH at 2–4 WAT but later experiencing pest resurgence, possibly due to disruptions in natural enemy dynamics caused by insecticide use. In contrast, T1 consistently maintained higher populations of spiders and *Micraspis* sp., indicating that EE supports a robust biological control system. These findings are consistent with those of Ali et al. [1, 61], Horgan et al. [45], and Gurr et al. [66], who emphasized that EE can strengthen agroecosystem resilience by conserving natural enemies and reducing pesticide reliance. Importantly, the inclusion of a true untreated control (T3) and multiseason observations adds to the robustness of our conclusions. Compared to the study by Ibrahim and Mugiasih [42], which lacked a true untreated control and did not measure tungro incidence and rice yield, our study provides a more comprehensive evaluation of EE's effectiveness in pest and disease management.

Insecticide application in T2 appeared to reduce populations of beneficial insects, especially parasitoids, which were mostly from the family Ichneumonidae. Earlier studies have shown that *Zinnia* and *Tagetes* planted on bunds can support parasitoids from families such as Braconidae, Tachinidae, and Scelionidae [1, 42, 67]. Peñalver-Cruz and Horgan [68] found that planting nectar-rich bund vegetation alongside resistant rice varieties increased parasitism of planthopper eggs and reduced the need for insecticides.

Higher spider populations were consistently recorded in T1, where insecticides were not used. Spiders are known to be effective generalist predators in rice fields [69, 70]. Globally, spiders are estimated to consume between 400 and 800 million tonnes of insects annually, with Tetragnathidae being the most dominant spider family in rice fields [71, 72].

The reduced capture rates of GLH natural enemies in T2 further confirm that broad-spectrum insecticide application can diminish beneficial arthropods. Several previous studies have reported similar findings, indicating that such insecticide use negatively affects predator and parasitoid populations [67, 73–76]. Misuse or overuse of insecticides has also been linked to secondary pest outbreaks in rice ecosystems [1, 49, 77, 78]. Our study shows that rice plots with EE treatment (T1) had the highest numbers of natural enemies and the lowest pest incidence, while insecticide-treated plots (T2) had the fewest natural enemies. The untreated control (T3) supported more natural enemies than T2, but fewer than T1, reaffirming the effectiveness of habitat management and biopesticides [1, 45, 66].

Tungro suppression in T1 was likely due to a combination of reduced GLH vector populations and higher predation pressure from natural enemies (Figure 3). These findings align with previous reports demonstrating that EE can reduce vector-borne viral diseases by promoting biological suppression of vectors through predators and EPFs [79–81].

Interestingly, although pest and disease pressures varied, rice yields were not significantly different among treatments (*p* > 0.05) (Figure 4). This suggests that EE strategies can provide reliable pest and vector control without yield penalties. Such findings are critical, as farmers are often hesitant to adopt sustainable alternatives if they are perceived to reduce yields. Our results support earlier conclusions that biodiversity-based pest management does not compromise productivity [66, 82].

These results highlight the strong potential of EE as a sustainable alternative to conventional pesticide-based systems. It effectively enhances natural enemy populations, suppresses pest and vector incidence, and maintains crop yield. The comparable yields of T1 to those of T2 and T3, combined with its superior performance in reducing tungro and GLH, present a strong argument for its broader adoption. This study is one of the first in Indonesia to evaluate multiseason integration of flowering bund plants and biopesticides, offering a landscape-level perspective on sustainable rice pest management.

However, this study also had some inadequacy. Natural enemies were identified only at the group level, and the individual contributions of each flowering plant species were not assessed. Moreover, the study was conducted at a single location, which may limit the generalizability of the findings across different rice-growing regions. Future studies should aim to identify natural enemies at the species level, evaluate the specific roles of different bund plants, and conduct experiments across multiple locations. These efforts will refine EE strategies and enhance their applicability across diverse agroecological settings.

## 5. Conclusion

This study demonstrated that EE, involving the integration of nectar-rich flowering plants on rice bunds and the application of biopesticides composed of *M. anisopliae* and *A. paniculata* extract, can effectively suppress GLH populations and reduce tungro virus incidence while maintaining rice yield. The EE treatment (T1) consistently supported higher populations of natural enemies—such as spiders, parasitoids, and predatory beetles—compared to the insecticide-treated (T2) and untreated control (T3) plots. Tungro incidence was lowest in T1 and highest in T3, while rice yields did not differ significantly among treatments across the three dry seasons. These findings highlight the potential of habitat manipulation through EE as a sustainable alternative to chemical pest control in rice ecosystems. In Indonesia, rice fields are typically divided into small plots with distinct bunds, offering spatial opportunities for the adoption of this approach. Future research should focus on identifying natural enemies at the species level, evaluating the contribution of individual flowering plant species, and conducting multilocation field trials to improve the robustness, scalability, and practical applicability of EE strategies in diverse rice-growing regions.

## Data Availability

The data that support the findings of this study are available from the corresponding author upon reasonable request.
